# The semaphorin 3A/neuropilin-1 pathway promotes clonogenic growth of glioblastoma via activation of TGF-**β** signaling

**DOI:** 10.1172/jci.insight.167049

**Published:** 2023-11-08

**Authors:** Hye-Min Jeon, Yong Jae Shin, Jaehyun Lee, Nakho Chang, Dong-Hun Woo, Won Jun Lee, Dayna Nguyen, Wonyoung Kang, Hee Jin Cho, Heekyoung Yang, Jin-Ku Lee, Jason K. Sa, Yeri Lee, Dong Geon Kim, Benjamin W. Purow, Yeup Yoon, Do-Hyun Nam, Jeongwu Lee

**Affiliations:** 1 Department of Cancer Biology, Lerner Research Institute, Cleveland Clinic, Cleveland, Ohio, USA.; 2 Institute for Refractory Cancer Research, Samsung Medical Center, Seoul, South Korea.; 3 Graduate School of Health Science & Technology, Samsung Advanced Institute for Health Science & Technology, Sungkyunkwan University, Seoul, South Korea.; 4 Department of Biomedical Convergence Science and Technology, Kyungpook National University, Daegu, South Korea.; 5 Department of Biomedical Sciences and Department of Anatomy and Cell Biology, Seoul National University, College of Medicine, Seoul, South Korea.; 6 Department of Biomedical Sciences, Korea University, College of Medicine, Seoul, South Korea.; 7 Department of Neurology, University of Virginia, Charlottesville, Virginia, USA.

**Keywords:** Development, Oncology, Brain cancer, Growth factors, Oncogenes

## Abstract

Glioblastoma (GBM) is the most lethal brain cancer with a dismal prognosis. Stem-like GBM cells (GSCs) are a major driver of GBM propagation and recurrence; thus, understanding the molecular mechanisms that promote GSCs may lead to effective therapeutic approaches. Through in vitro clonogenic growth-based assays, we determined mitogenic activities of the ligand molecules that are implicated in neural development. We have identified that semaphorin 3A (Sema3A), originally known as an axon guidance molecule in the CNS, promotes clonogenic growth of GBM cells but not normal neural progenitor cells (NPCs). Mechanistically, Sema3A binds to its receptor neuropilin-1 (NRP1) and facilitates an interaction between NRP1 and TGF-β receptor 1 (TGF-βR1), which in turn leads to activation of canonical TGF-β signaling in both GSCs and NPCs. TGF-β signaling enhances self-renewal and survival of GBM tumors through induction of key stem cell factors, but it evokes cytostatic responses in NPCs. Blockage of the Sema3A/NRP1 axis via shRNA-mediated knockdown of Sema3A or NRP1 impeded clonogenic growth and TGF-β pathway activity in GSCs and inhibited tumor growth in vivo. Taken together, these findings suggest that the Sema3A/NRP1/TGF-βR1 signaling axis is a critical regulator of GSC propagation and a potential therapeutic target for GBM.

## Graphical abstract

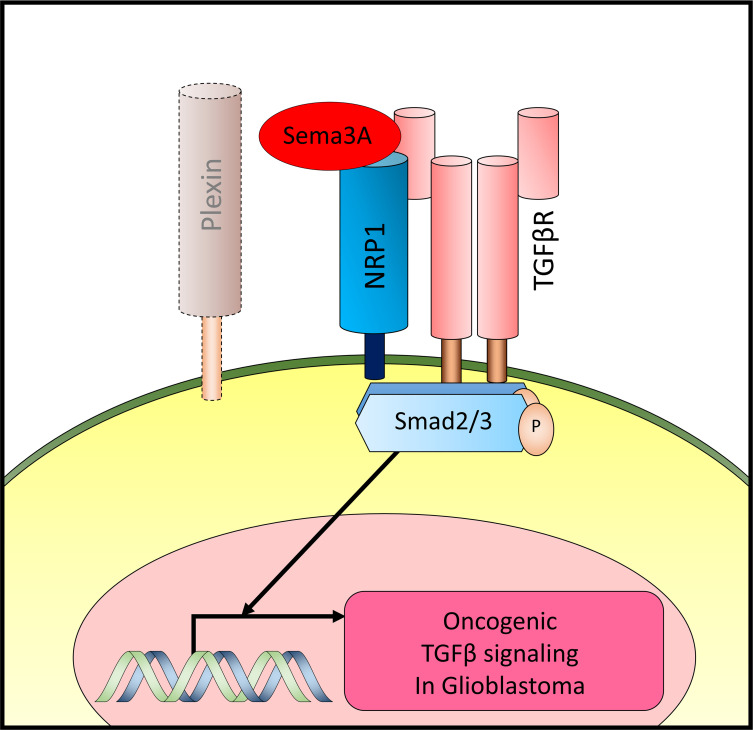

## Introduction

Glioblastoma (GBM) is the most common and the most lethal brain tumor in adults. Median survival of patients with GBM who have received the standard-of-care treatments, tumor resection followed by radiotherapy plus concomitant and adjuvant temozolomide, is only 14 to 15 months (1). Pharmacological efforts to target specific RTKs, such as EGFR and MET; antiangiogenic therapies; and recent immunotherapy-based approaches have not yet demonstrated prominent therapeutic benefits for a large cohort of patients with GBM, emphasizing an urgent need for novel, effective anti-GBM therapeutic approaches (2).

GBM tumors display profound heterogeneity in genetic and epigenetic landscapes, and these states are highly plastic and dynamic. GBM stem-like cells (GSCs) are a critical cell population that drives GBM propagation and recurrence (3, 4). GSCs share numerous phenotypic similarities with normal neural stem/progenitor cells (NPCs), including the expression of cell surface marker proteins, close physical proximity to blood vessels, and regulation of stem cell signaling pathways. Recent therapeutic approaches to target the core stem cell pathways, such as Notch, Wnt, and Hedgehog, have been extensively investigated (5–10). While the preclinical data from multiple studies underscore the promise of these therapeutic approaches, there exists a potential concern of normal cell toxicity since the above pathways play critical roles in normal cells as well.

It is increasingly clear that various neurotransmitters, neurotrophins, synaptic adhesion molecules, and axon guidance molecules, previously thought to regulate CNS functions, can promote CNS tumor initiation and progression. Recent studies have shown that nerve growth factor (NGF), neurotrophins (NTs), synaptic adhesion molecule neuroligin, and brain-derived neurotrophic factor (BDNF) promote the initiation, progression, and invasion of gliomas (11–16). Similarly, various axon guidance molecules, such as Netrin, Slit, Ephrin, Nogo, and semaphorins, play critical roles not only in CNS development but also in cancer and the tumor microenvironment (11–17). However, whether these molecules have direct mitogenic effect on GSCs and their effects are differentially regulated in GSCs compared with NPCs are largely unknown.

In this study, we have utilized a series of recombinant proteins that are known to be involved in brain developmental programs and screened for their proliferative/clonogenic effects on GSCs in comparison with normal NPCs. We found that semaphorin 3A (Sema3A) is a potent mitogen for GSCs via activation of canonical TGF-β signaling. We then provide a molecular basis of the Sema3A/neuropilin-1/TGF-β receptor type 1 (Sema3A/NRP1/TGF-βR1) signaling axis in GBM and propose that this signaling node is a key GSC regulator and a potential therapeutic target.

## Results

### Sema3A-induced GBM proliferation is mediated by NRP1.

GSCs and NPCs share common regulatory mechanisms of self-renewal, including well-known mitogens such as EGF, FGF2, and HGF. We hypothesized that some ligands that are traditionally implicated in neural functions may harbor previously unrecognized mitogenic and/or clonogenic capacities for GSCs and/or NPCs. As an initial screen to identify such molecules, we chose 24 ligand molecules that are critical regulators of synaptic adhesion, axon guidance, and neurotropic functions (Supplemental Table 1; supplemental material available online with this article; https://doi.org/10.1172/jci.insight.167049DS1). We have utilized 2 patient-derived primary GBM lines (131 and 827) and 2 different NPCs derived from fetal brain tissues (NPC1) or human embryonic stem cells (NPC2). These cells were plated at varying seeding densities in serum-free media, then cultured in the presence of the recombinant proteins of interest (R&D Systems), and their growth was then evaluated 2 weeks later. Well-known mitogens such as EGF and FGF2 were used as positive controls. A few of the recombinant proteins, such as NGF and BDNF, enhanced survival and growth of both GSCs and NPCs. Notably, recombinant Sema3A protein (rSema3A) was a top hit in our screen, which enhanced the growth of GSCs, particularly 131 mesenchymal GBM lines. In contrast, 2 different normal NPCs treated with rSema3A showed slightly decreased proliferation compared with the control (Supplemental Table 1).

**Figure 1:**
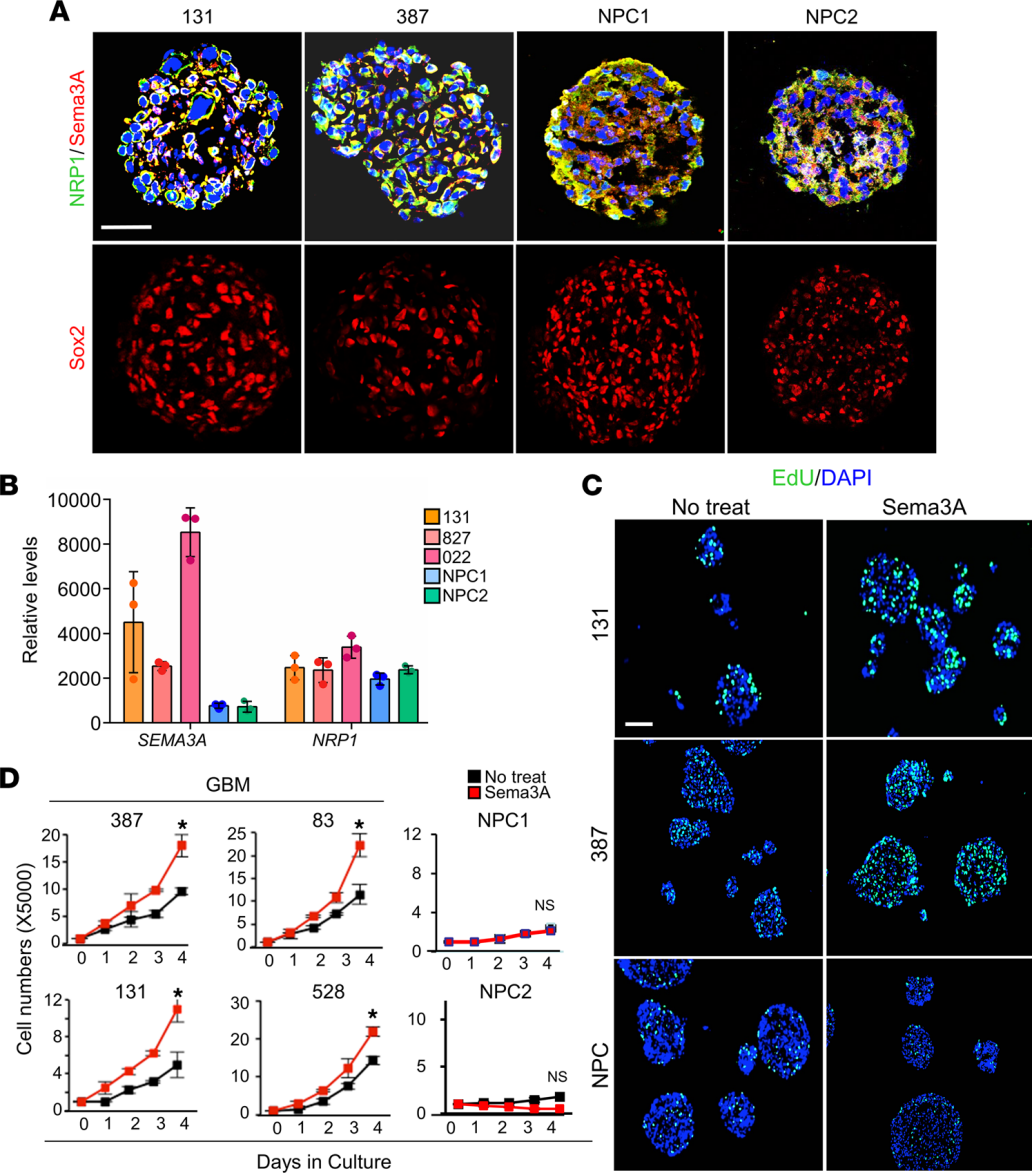
Sema3A enhances GBM proliferation. (**A**) IF images of Sema3A, NRP1, and SRY-box transcription factor 2 (Sox2) in patient-derived 131 and 387 GBM and NPC-derived spheres. (**B**) Levels of *Sema3A* and *NRP1* mRNA in 131, 827, and 022 GBM cells and NPCs. *n* = 3. (**C**) Representative images of EdU incorporation assays using GBM cells and NPCs treated with or without Sema3A (10 ng/mL). Cells labeled with green color are EdU-positive cells. (**D**) Proliferation assays to determine the effect of rSema3A on growth of GBM cells and NPCs. *n* = 3. *P < 0.01 by 1-way ANOVA. Data represent mean ± SD. Scale bars: 50 μm.

Semaphorins make up the largest family of axon guidance cues. Sema3A is classically described as a collapsing factor and a mediator of axon repulsion, but its roles have been extended to tumor growth, invasion, and angiogenesis (18). Neuropilins and plexins are well-known receptor families for Sema3A (19, 20). We found that primary GBM cells and NPCs expressed both Sema3A and NRP1, as determined by immunofluorescence (IF) staining and RNA-Seq analysis (Figure 1, A and B). Next, we determined whether rSema3A-induced GBM proliferation is broadly applicable to various GBM tumors. Compared with nontreated controls, rSema3A treatment significantly increased the number of 5-ethynyl-2′-deoxyuridine–positive (EdU-positive) cells and the total cell counts in GBM cells derived from 6 different patients (Figure 1, C and D). Neurosphere-like clonogenic cell growth is an in vitro indicator of GSC self-renewal (21). Neurospheres were much more readily formed in rSema3A-treated wells compared with the untreated control, as determined by neurosphere formation limiting dilution assays (Supplemental Figure 1A). In sharp contrast, rSema3A treatment did not enhance proliferation of 3 different NPCs derived from fetal brain tissues, induced pluripotent stem cells, and human embryonic stem cells (Figure 1, C and D, and data not shown). These data suggest that Sema3A may selectively induce proliferation of a broad range of GBM cells but not NPCs.

**Figure 2:**
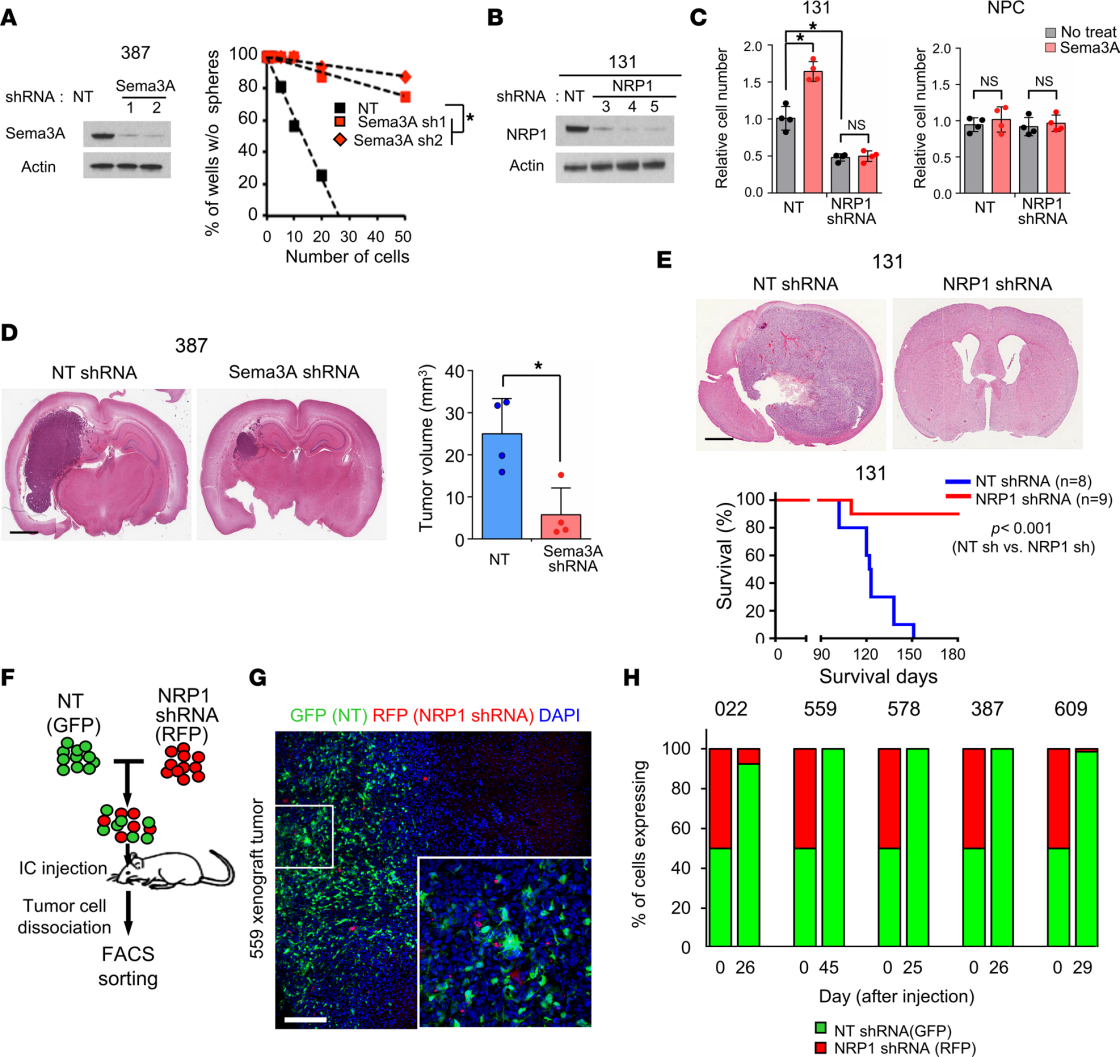
Sema3A promotes clonogenic growth of GBM cells via NRP1. (**A**) Left, IBs of Sema3A in 387 GBM cells transduced with either NT control or *Sema3A* shRNA-expressing lentivirus. β-Actin was used as a loading control. Right, limiting dilution assay (LDA) analysis to determine clonogenic growth of GBM cells with NT or *Sema3A* shRNAs. *P < 0.01 by pairwise *t* test. (**B** and **C**) IBs of NRP1 expression and proliferation index in 131 GBM cells transduced with either NT control or *NRP1* shRNA-expressing lentivirus. *n* = 4. *P < 0.01 by 1-way ANOVA with Tukey’s multiple-comparison test. (**D**) Left, representative H&E brain sections of the mice that were injected with either NT or *Sema3A* KD cells. Right, quantitation of tumor volumes in the brain sections. *n* = 4. *P < 0.01 by unpaired, 1-tailed Student’s *t* test. (**E**) Top, representative H&E brain sections of the mice that were injected with either NT or *NRP1*-KD 131 cells. Bottom, Kaplan-Meier survival curves of mice orthotopically implanted with 131 cells transduced with either NT shRNA– (*n* = 8) or *NRP1* shRNA-expressing lentivirus (*n* = 9). *P < 0.001 by log-rank test. (**F**) Scheme of in vivo tumor growth competition assay. Equal numbers of NT shRNA–expressing GBM cells (GFP-labeled) and *NRP1* shRNA–expressing GBM cells (RFP-labeled) were mixed and injected into the brains of mice. The resultant tumors were dissociated into single cells and processed for FACS analysis. (**G**) Representative images of 559 xenograft tumor. Inset shows a high-power image. (**H**) Quantitation of GBM tumors derived from the mixture of NT shRNA– and *NRP1* shRNA–expressing cells. Tumors were harvested when the animals showed neurological signs, and tumor latency per each tumor is indicated in *x* axis. Data represent mean ± SD. Scale bars: 2 mm (**D** and **E**), 50 μm (**G**).

Expression of Sema3A and NRP1 in GSCs suggests that Sema3A is a ligand for GBM-autonomous proliferative signaling. shRNA-mediated knockdown of either *Sema3A* or *NRP1* significantly reduced clonogenic growth of GSCs (Figure 2, A–C, and Supplemental Figure 1B). Furthermore, rSema3A did not induce proliferation in *NRP1*-knockdown GBM cells, indicating that Sema3A increases clonogenic growth of GBM cells in an *NRP1*-dependent manner (Figure 2C). Unlike GSCs, NRP1 knockdown did not affect survival and proliferation of NPCs (Figure 2C).

We assessed the effects of *Sema3A* or *NRP1* knockdown on glioma-forming ability in vivo (Figure 2, D–H). We transduced GBM cells with lentiviruses expressing either nontargeting (NT) shRNA or *Sema3A* shRNA, implanted these cells into the brains of nude mice, and then harvested the brains 20 days later. Resultant tumors derived from *Sema3A*-knockdown 387 GBM cells were significantly smaller than those from the control (Figure 2D). Notably, *NRP1* knockdown showed much more robust antitumor effects. While most animals bearing NT shRNA-expressing 131 GBM cells died within 4 months (median survival: 123 days), animals injected with *NRP1* shRNA-expressing cells survived significantly longer with no visible tumors (*P* < 0.001) (Figure 2E). To determine whether NRP1 targeting can elicit strong antitumor effects in a broad range of GBMs, we performed in vivo growth competition assays in which control (GFP-labeled) or *NRP1* shRNA (RFP-labeled) tumor cells were mixed in a 1:1 ratio and coinjected into mouse brains. We harvested the resulting tumors and performed FACS analysis and histological analysis (Figure 2, F–H). In all 5 different GBM tumors, more than 95% of the resulting tumor cells were derived from GFP-positive, control shRNA-expressing cells.

### The Sema3A/NRP1 axis activates canonical TGF-β pathway in GBM.

The above data indicate the mitogenic/clonogenic role of Sema3A/NRP1 signaling in GBM but not in NPCs. NRP1, a membrane-bound coreceptor, is known to interact with multiple cell surface receptor proteins, including VEGF receptor 2 (VEGFR2) (22, 23), MET (24), and TGF-βR1 (25). Notably, it has been well established that TGF-β signaling enhances self-renewal, invasiveness, and the tumorigenic potential of GBM tumors through induction of key stem cell factors such as leukemia inhibitory factor (LIF), inhibitor of DNA-binding proteins (IDs), and SOX family proteins (26–28). In NPCs, however, TGF-β signaling evokes a cytostatic response or neural differentiation by inducing the expression of p15^ink4b^ and p21^Cip1^ and by suppressing IDs (29, 30). These functional dichotomic downstream effects by TGF-β signaling in the context of GBM and normal neural cell counterparts are one of the well-known examples of the “TGF-β paradox.” Thus, we hypothesized that the Sema3A/NRP1 signaling in GBM cells and NPCs induces TGF-β pathway activation.

**Figure 3:**
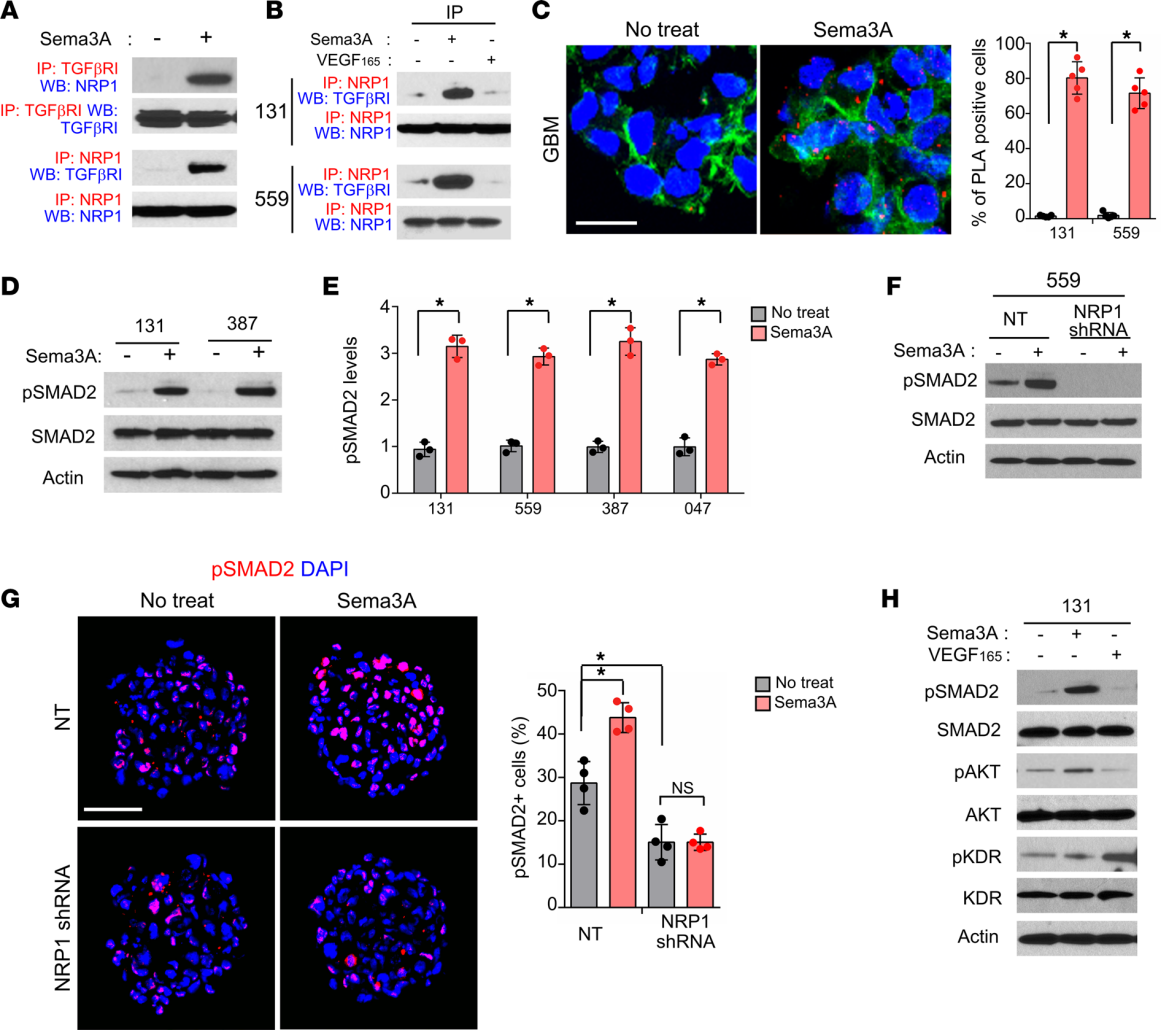
Sema3A activates TGF-β signaling via NRP1-TGF-βR1 interaction. (**A**) Co-IP IBs of NRP1 and TGF-βR1 in 131 GBM cells treated with rSema3A. For IP-IB data, Abs used for IP and Western blotting (WB) are labeled as red and blue, respectively. (**B**) Co-IP IBs of NRP1 and TGF-βRI in GBM cells treated with rSema3A or rVEGF_165_. (**C**) Left, representative images of PLAs using anti-NRP1 and anti–TGF-βR1 Abs. Red dots represent the positive signal due to the proximity of 2 added Abs. Alexa Fluor 488–conjugated phalloidin (green) and DAPI (blue) were used to visualize actin cytoskeleton and nuclei, respectively. Right, red dots were counted in 5 random fields and plotted. *n* = 5. Scale bar: 20 μm. (**D** and **E**) IBs of p-SMAD2 and total SMAD2 expression in GBM cells treated with or without rSema3A. *n* = 3. (**F**) IBs of p-SMAD2 in the NT control and *NRP1*-knockdown 559 GBM cells treated with or without rSema3A. (**G**) Immunostaining and quantitation of p-SMAD2 in the NT control and *NRP1*-knockdown 131 and 559 GBM cells treated with or without rSema3A. Scale bar: 50 μm. *n* = 4. (**H**) IBs of p-SMAD2, total SMAD2, p-KDR, and total KDR in 131 GBM cells treated with rSema3A (50 ng/mL) and rVEGF_165_ (100 ng/mL). Data represent mean ± SD. *P < 0.01 by 1-way ANOVA in **C** and **E**. *P < 0.01 by 1-way ANOVA with Tukey’s multiple-comparison test in **G**.

Co-IP experiments using anti-NRP1 Ab revealed that NRP1 coprecipitated with TGF-βR1 in protein lysates isolated from GBM cells (131, 559, and 83) and specimens of patients with GBM (047 and 050) (Supplemental Figure 2, A and B). Notably, rSema3A treatment robustly increased the level of NRP1–TGF-βR1 complexes (Figure 3, A and B). As an alternative and complementary approach to verify molecular interaction between NRP1 and TGF-βR1 proteins in GBM cells, we performed proximity ligation assays (PLAs) that enable the visualization of protein-protein interactions in cells (Figure 3C). Consistent with IP data, NRP1–TGF-βR1 interaction in GBM cells was significantly increased after treatment with rSema3A, as demonstrated by the increased numbers of PLA spots (Figure 3C). We then hypothesized that NRP1–TGF-βR1 interaction may lead to activation of canonical TGF-β signaling, in which SMAD2 is an immediate downstream effector. Indeed, treatment with rSema3A rapidly increased the levels of SMAD2 phosphorylation in 4 different GBM lines and 2 NPCs (Figure 3, D and E, and Supplemental Figure 2C). In contrast, Sema3A-triggered phosphorylated SMAD2 (p-SMAD2) activation was almost completely blocked in *NRP1*-knockdown GBM cells (Figure 3, F and G).

As NRP1 is also implicated in VEGF-dependent VEGFR2 (also called KDR) signaling in GBM (31), we examined whether VEGF could induce TGF-β signaling activation in these cells similar to Sema3A. VEGF increased phosphorylation of KDR but not SMAD2 in 131 GBM cells (Figure 3H). In contrast, Sema3A increased the levels of p-SMAD2 without affecting KDR phosphorylation, suggesting that Sema3A and VEGF signaling act independently in these cells. Finally, to further verify whether NRP1–TGF-βR1 interaction requires Sema3A, we overexpressed the WT *NRP1* or mutant *NRP1* that is devoid of Sema3A binding domain (Supplemental Figure 2D). While ectopic expression of the WT *NRP1* in 387 GBM cells further increased p-SMAD2 and rSema3A-induced proliferation, mutant *NRP1* expression had no or negative effects (Supplemental Figure 2, E and F). Together, these data suggest that Sema3A activates TGF-β signaling in GBM cells by engaging in interaction between NRP1 and TGF-βR1.

**Figure 4:**
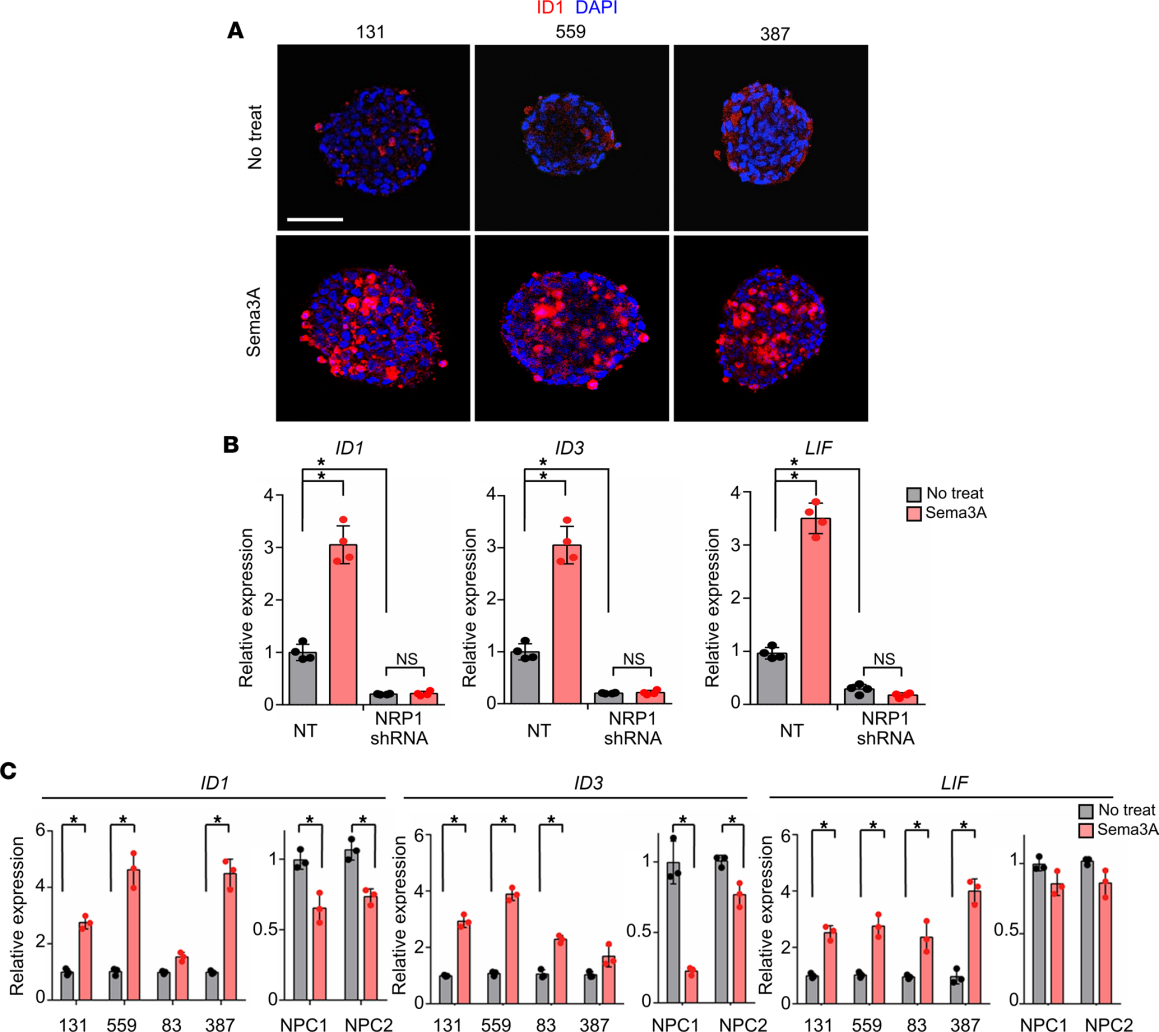
The Sema3A/NRP1 axis in GBM activates canonical TGF-β signaling. (**A**) Immunostaining images of ID1 in 131, 559, and 387 GBM cells treated with rSema3A. ID1-positive cells are shown in red. Scale bar: 50 μm. (**B**) Levels of the representative TGF-β pathway genes (*ID1*, *ID3*, and *LIF*) in the NT or *NRP1*-KD 131 GBM cells. *n* = 4. (**C**) Levels of *ID1*, *ID3*, and *LIF* mRNAs in GBM cells and NPCs treated with rSema3A. *n* = 3. Data represent mean ± SD. *P < 0.01 by 1-way ANOVA with Tukey’s multiple comparison test in **B** and **C**.

We postulated that dichotomic responses of GSCs and NPCs to Sema3A are due to differential effects of TGF-β signaling activation. We found that rSema3A robustly induced expression of ID1, a key downstream factor of TGF-β signaling, in GBM cells (Figure 4A). Furthermore, mRNA levels of TGF-β target genes (*ID1*, *ID3*, and *LIF*) in GBM cells were significantly increased by rSema3A, and this induction was abolished in *NRP1*-knockdown GBM cells, suggesting that NRP1 is a main effector molecule in Sema3A-induced TGF-β signaling activation (Figure 4B). Last, we validated the expression of the above genes in additional GBM cells and NPCs. In 4 different GBM cells, expression levels of *LIF*, *ID1*, and *ID3* were significantly increased by rSema3A compared with nontreated controls. In sharp contrast, rSema3A treatment failed to induce mRNA levels of these genes in NPCs (Figure 4C).

**Figure 5:**
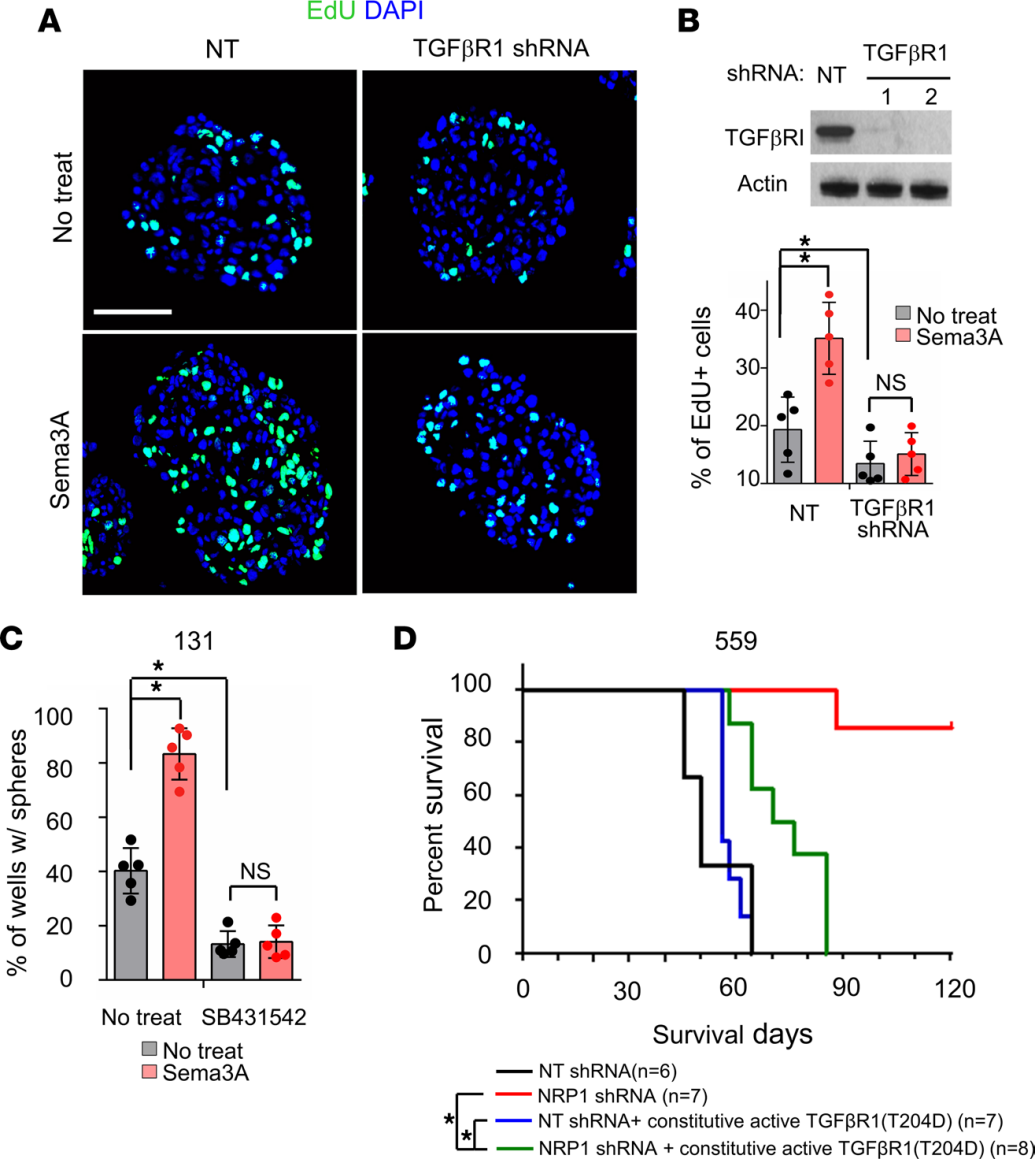
TGF-βR1 is a key downstream mediator of Sema3A/NRP1 signaling. (**A** and **B**) Representative immunostaining images and quantitation of EdU-positive cells in NT (control) and *TGF-βRI*–KD GBM cells treated with rSema3A. *TGF-βRI* KD was confirmed by IB analysis. Scale bar: 50 μm. *n* = 5. (**C**) LDA of 131 GBM cells treated with a TGF-βR inhibitor, SB431542 (2 μM), and rSema3A. *n* = 5. (**D**) Kaplan-Meier survival curves of mice orthotopically implanted with 559 GBM cells transduced with NT shRNA (control, *n* = 6), *NRP1* shRNA (*n* = 7), NT shRNA + T204D TGF-βR1 mutant (*n* = 7), and *NRP1* shRNA + T204D TGF-βR1 mutant (*n* = 8). *P < 0.001 by log-rank test. Data represent mean ± SD. *P < 0.01 by 1-way ANOVA with Tukey’s multiple-comparison test in **B** and **C**.

The above data collectively support that canonical TGF-β pathway activation is a downstream event of Sema3A/NRP1 signaling in GBM cells. To determine the extent of TGF-β signaling in Sema3A-mediated GBM proliferation/survival, we performed functional blocking and/or rescue experiments. First, we transduced 131 GBM cells with lentivirus-expressing shRNA against *TGF-βR1* and assessed biological effects on these cells. TGF-βR1 knockdown significantly diminished both Sema3A-induced proliferation and clonogenic growth of GBM cells, suggesting that TGF-β pathway is a key downstream effector of the Sema3A/NRP1 signaling (Figure 5, A and B). Second, we treated GBM cells with SB-431542, a small molecule inhibitor of TGF-βR1, and assessed the effects of rSema3A on these cells. Similar to *TGF-βR1* knockdown, SB-431542 treatment impaired Sema3A-induced proliferation and clonogenic growth of 131 GBM cells (Figure 5C). Conversely, ectopic expression of TGF-βR1 T204D protein, a constitutively active *TGF-βR1* mutant, increased SMAD2 phosphorylation and clonogenic growth of GBM cells (Supplemental Figure 3, A–C). Finally, to verify whether TGF-β signaling is a key downstream mediator of NRP1 signaling in GBM, we performed in vivo rescue experiments (Figure 5D). Whereas mice injected with *NRP1*-knockdown GBM cells survived significantly longer than the control, ectopic expression of TGF-βR1 T204D in *NRP1*-knockdown GBM cells restored tumor formation capacity (Figure 5D). Taken together, these results strongly support the notion that the Sema3A/NRP1 axis activates oncogenic TGF-β signaling circuit in GBM.

### NRP1^hi^ GBM cells are enriched with clonogenicity and TGF-β activity.

**Figure 6:**
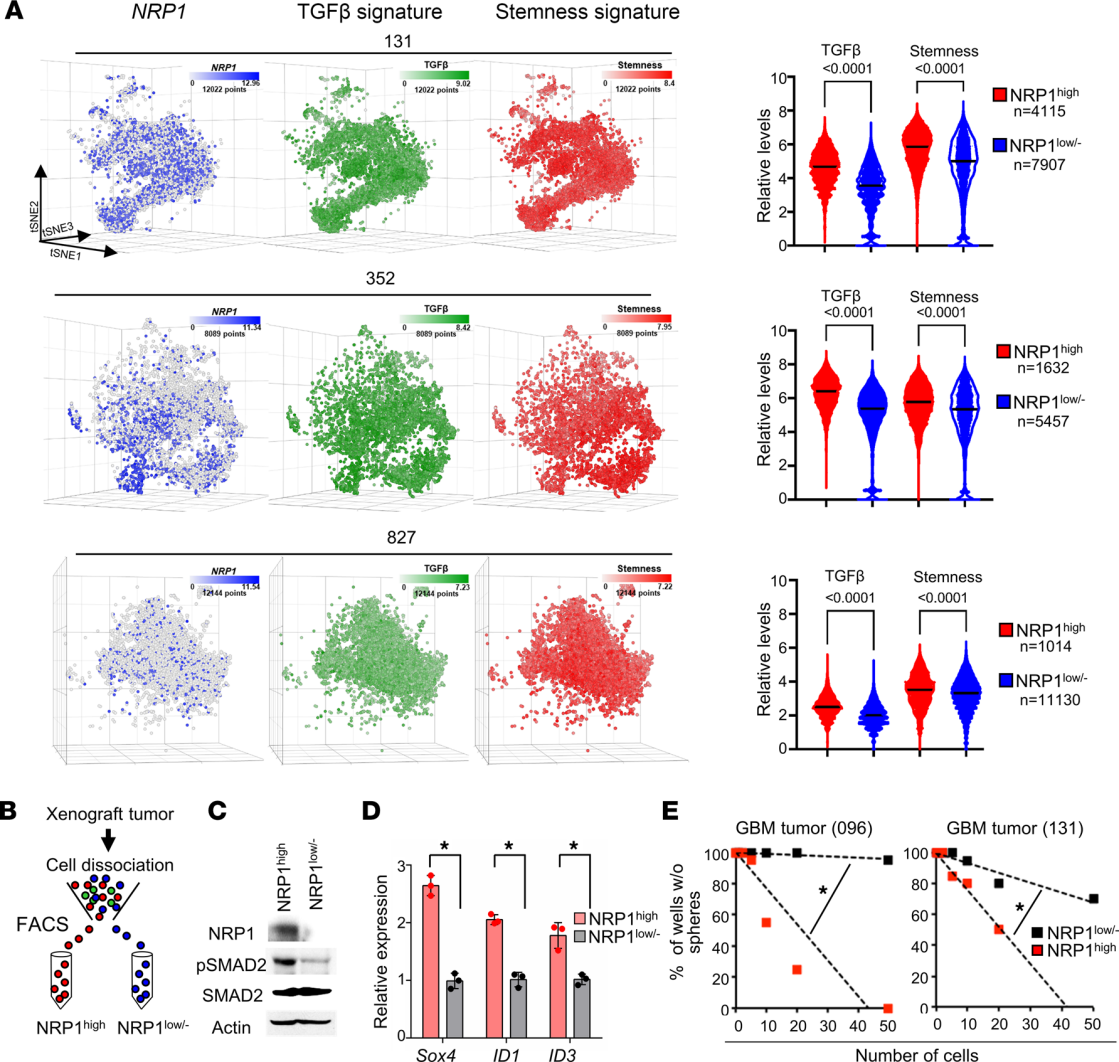
NRP1^hi^ GBM cells are enriched with clonogenicity and TGF-β activity. (**A**) Left, *t*-distributed stochastic neighbor embedding (t-SNE) plots of GBM single cells (131, 352, and 827; total of 31,255 cells). Color gradient was overlaid with *NRP1* (blue), TGF-β (green), and stemness (red) signature scores. Right, quantitation of TGF-β and stemness gene set expression in NRP1^hi^ cells and NRP1^lo/–^ GBM cells: 131, 352, and 827. Internal line represents median value. *P < 0.0001 by Mann-Whitney test. (**B**) FACS-based segregation of NRP1^hi^ and NRP1^lo/–^ cells from GBM tumors. (**C**) IBs of NRP1, p-SMAD2, and SMAD2 in NRP1^hi^ and NRP1^lo/–^ cells derived from primary GBM tumor 096. (**D**) Relative levels of Sox4, ID1, and ID3 in NRP1^hi^ cells and NRP1^lo/–^ GBM cells (096). Data represent mean ± SD. *P < 0.01 by 1-way ANOVA. (**E**) LDA analysis of NRP1^hi^ and NRP1^lo/–^ cells derived from GBM tumors (096 and 131). Estimated frequency of clonogenic cells in each subpopulation was calculated by extreme LDA. *P < 0.01 by pairwise *t* test.

The above data demonstrate the possibility that the Sema3A/NRP1 signaling axis influences cellular hierarchy in GBM in situ. To test, we performed single-cell RNA sequencing (scRNA-Seq) using 3 patient-derived GBM tumors and classified these cells based on the expression levels of *NRP1* mRNA, stemness, and TGF-β signaling gene signatures (32–36). NRP1^+^ subpopulation showed significantly higher levels of both stemness and TGF-β signaling gene signatures compared with matched NRP^–^ cells (Figure 6A).

To further verify, we isolated NRP1^hi^ and NRP1^lo/–^ subpopulations from GBM xenografts (096, 131, and 387) by FACS using an Ab that recognizes the extracellular domain of NRP1 (Figure 6B). NRP1^hi^ cells were operationally defined as the top 20% of NRP1-positive cells. NRP1^hi^ cells isolated from GBM tumors expressed higher levels of p-SMAD2 and TGF-β downstream target genes compared with matched NRP1^lo/–^ cells, indicating the elevated TGF-β signaling activation in NRP1^hi^ cells (Figure 6, C and D). Next, we determined the growth kinetics of each subpopulation after confirming the purity and viability of NRP1^hi^ and NRP1^lo/–^ populations. NRP1^hi^ cells proliferated more efficiently than NRP1^lo/–^ cells in the presence of rSema3A. Frequencies of neurosphere-forming clonogenic cells were significantly higher in NRP1^hi^ cells as compared with NRP1^lo/–^ cells, indicating that NRP1^hi^ cells harbor the enriched clonogenic capacity (Figure 6E).

**Figure 7:**
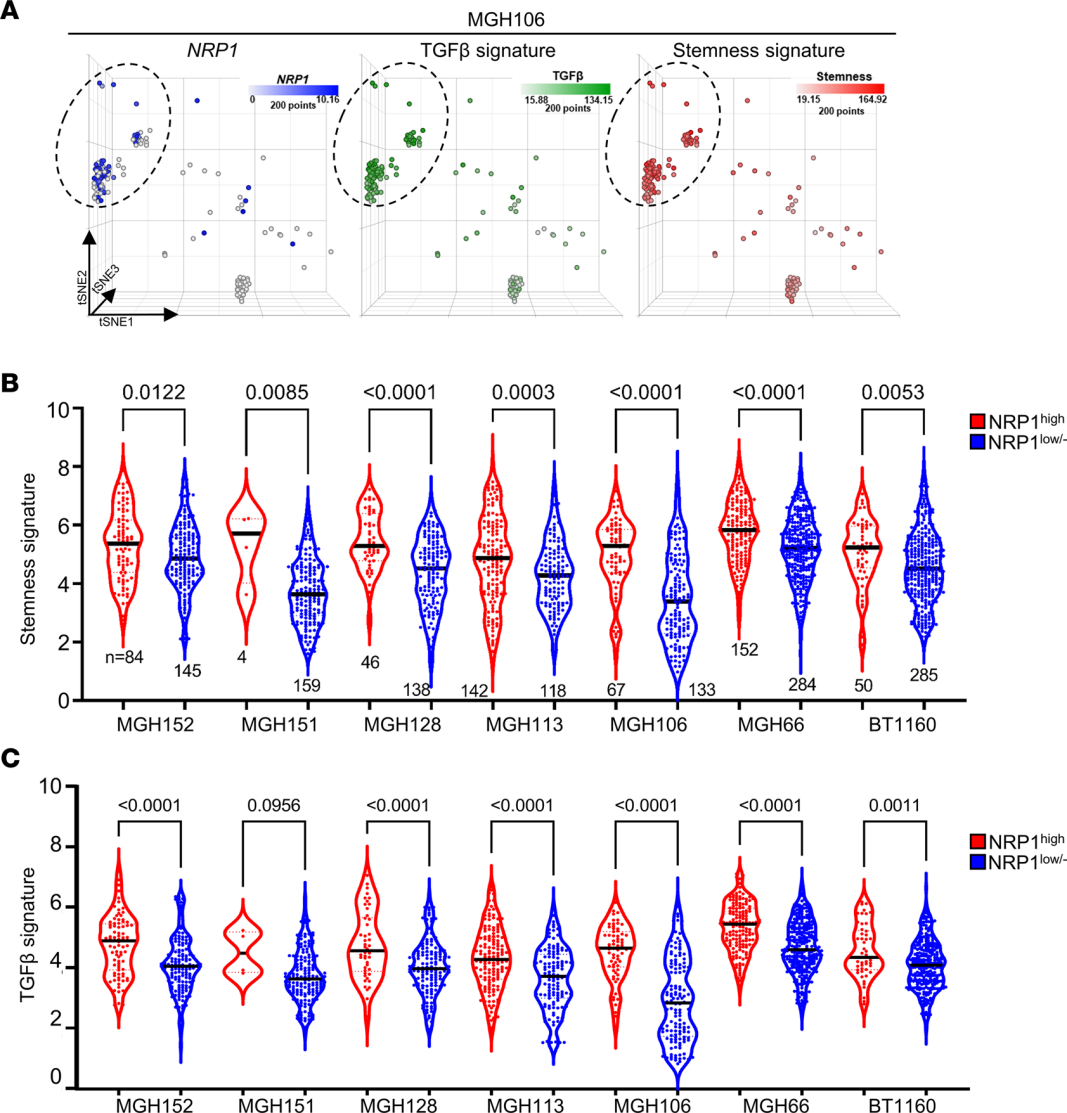
NRP1^hi^ cells are enriched with stemness and TGF-β signatures in primary specimens of patients with GBM. (**A**) t-SNE plots of tumor of patient with MGH106. Color gradient was overlaid with *NRP1* (blue), TGF-β (green), and stemness (red) signature scores. (**B** and **C**) Relative levels of the stemness and TGF-β signaling scores in the NRP1^hi^ and matched NRP1^lo/–^ subpopulations derived from 7 different tumors of patients with GBM. Internal line represents median value. *P* values were determined by Mann-Whitney test.

To validate clinical relevance of our findings in multiple human GBM specimens, we performed similar bioinformatics analyses using the publicly available GBM single-cell sequencing data sets. Based on relative levels of *NRP1* mRNA, we divided single cells into NRP1^hi^ and the remaining (NRP1^lo/–^) groups and determined relative levels of stemness and TGF-β signaling gene signatures in these subpopulations. NRP1^hi^ cells derived from 7 different tumors of patients with GBM revealed significantly enriched scores for stemness and TGF-β signaling compared with matched NRP1^lo/–^ cells (Figure 7, A–C).

### Expression of the Sema3A/NRP1 signaling components in GBM specimens.

**Figure 8:**
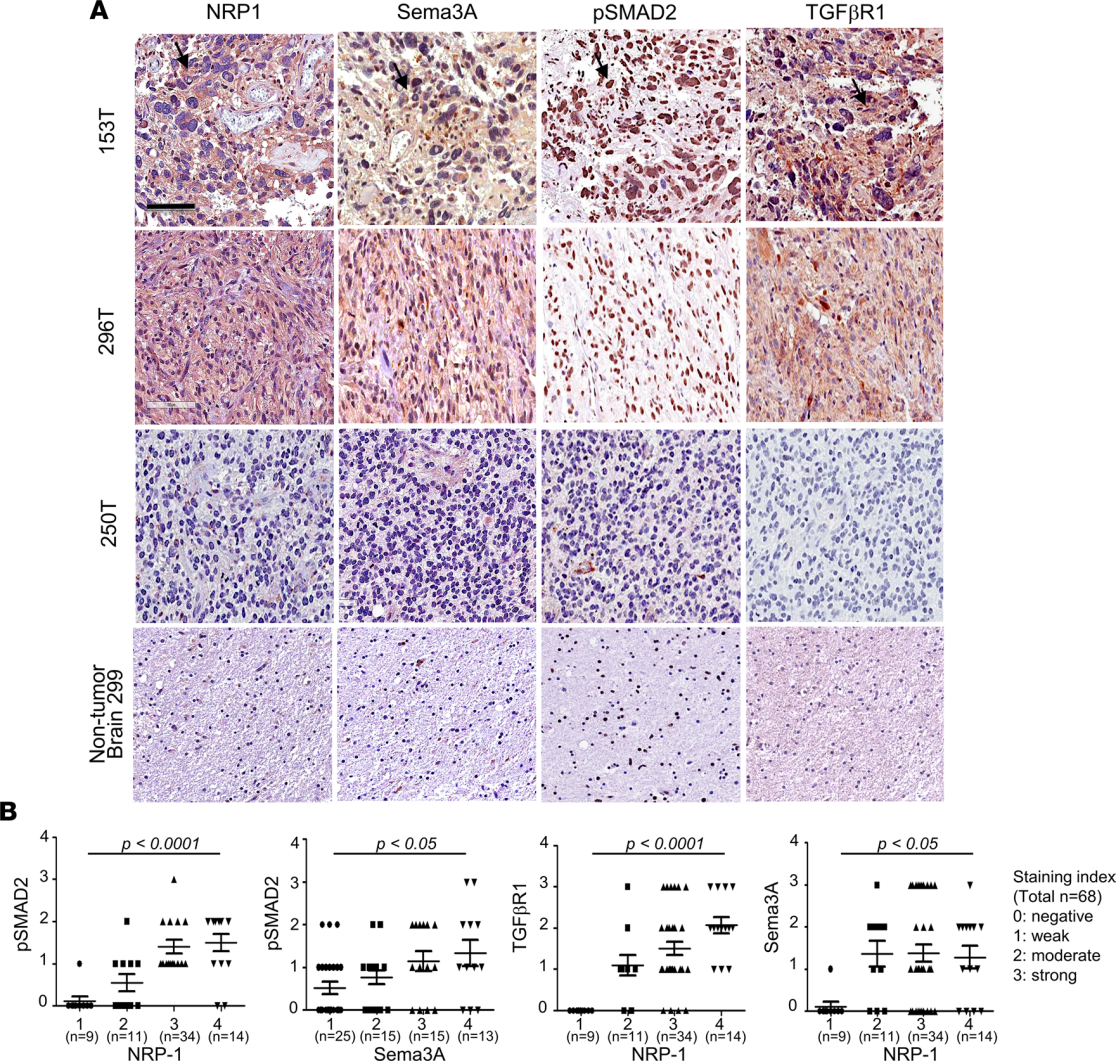
Expression of Sema3A/NRP1 signaling components in GBM. (**A**) Representative IHC images of NRP1, Sema3A, p-SMAD2, and TGF-βR1 using 3 GBM specimens — 153, 296, and 250 — and a nontumor brain tissue. Brown staining indicates immunopositivity for the indicated Ab. Scale bar: 100 μm. (**B**) Correlations between the expression levels of each of the Sema3A/NRP1 signaling components. Staining intensity of each IHC using Abs against NRP1, Sema3A, p-SMAD2, and TGF-βR1 in GBM specimens (*n* = 68) was determined and grouped: 0, negative; 1, weak; 2, moderate; 3, strong; and 4, very strong. Data are represented as vertical scattered plots using GraphPad Prism. Average intensity and SD are shown for the indicated Ab. *P* values were obtained using 2-way ANOVA.

To further establish clinical relevance of the Sema3A/NRP1 axis in GBM, we determined the expression levels of each of the signaling components in GBM specimens by tissue microarray (TMA), in which 68 GBM specimens and 10 non–tumor-bearing brain tissues were included (Figure 8A). Sema3A protein was barely detectable in nontumor tissues. In contrast, over 60% of GBM specimens revealed strong immunopositive staining patterns for Sema3A. Consistent with this, NRP1 staining was detected in 85% of GBM specimens. Notably, GBM specimens staining positive for NRP1 were almost always positive for Sema3A. In addition, NRP1 levels in GBM specimens positively correlated with the levels of Sema3A, TGF-βR1, and p-SMAD2 (Figure 8B). In addition, mRNA expression levels of *NRP1* in glioma specimens positively correlated with those of *Sema3A* or TGF-βR1, as determined by The Cancer Genome Atlas (TCGA) data analysis (37, 38) (Supplemental Figure 4). These findings corroborate well with the notion that the Sema3A/NRP1/TGF-β axis is an oncogenic signaling node in GBM.

### Association between the levels of Sema3A/NRP1 and survival of patients with glioma.

**Figure 9:**
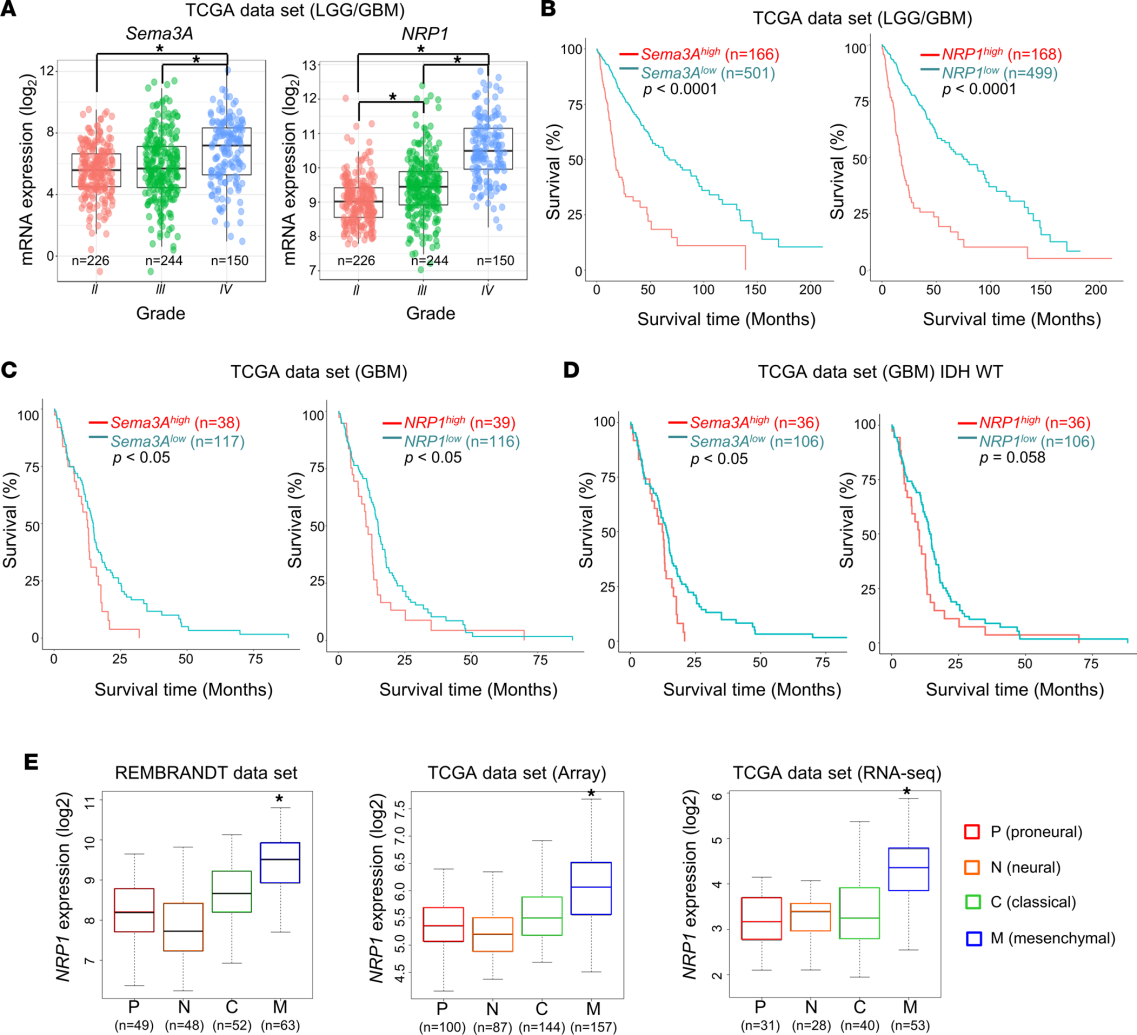
Association between Sema3A/NRP1 levels and survival of patients with glioma and GBM subtype. (**A**) Box-and-whisker plots showing mRNA levels of *Sema3A* and *NRP1* in LGG and GBM specimens in TCGA data set (*n* = 620). *P < 0.0001 by pairwise *t* test. (**B**) Kaplan-Meier survival curves of patients with LGG and GBM (*n* = 667) based on the expression levels of *Sema3A* or *NRP1* mRNA. High and low subgroups were operationally defined as the upper quartile (top 25%) and the rest, respectively. *P < 0.0001 by log-rank analysis. (**C** and **D**) Kaplan-Meier survival curves of patients with GBM and the WT IDH1/2-containing GBMs based on the levels of *Sema3A* or *NRP1* mRNA. *P < 0.05 by log-rank analysis. (**E**) Box-and-whisker plots of *NRP1* mRNA expression in 4 representative GBM subtypes. Repository of Molecular Brain Neoplasia Data (REMBRANDT) and TCGA (microarray data and RNA-Seq data sets, separately) databases were used to determine *NRP1* mRNA levels in each subtype of GBM. *P < 0.01 (mesenchymal subtype vs. other subtypes) by 1-way ANOVA with Tukey’s multiple-comparison test.

**Figure 10:**
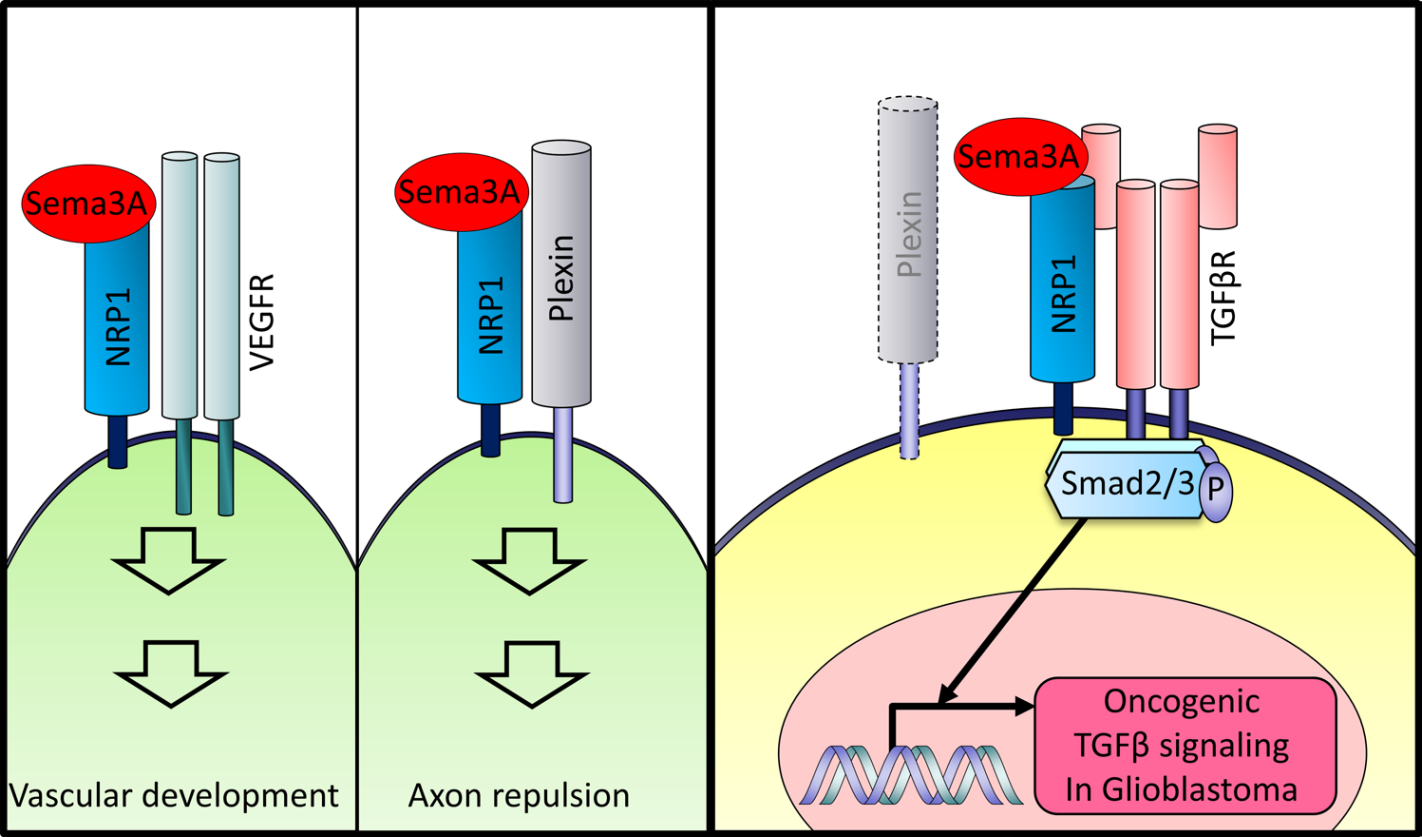
Roles of the Sema3A/NRP1 axis in developmental processes and GBM. Diagrams of the Sema3A and the associated protein complex in different biological programs and GBM.

To evaluate the potential correlation between the expression levels of the Sema3A/NRP1 axis components with tumor grade and survival, we interrogated TCGA clinical glioma data sets. The expression levels of *Sema3A* and *NRP1* strongly correlated with tumor grades, and they were significantly higher in GBMs compared with low-grade gliomas (LGGs) (Figure 9A). Based on mRNA levels of *Sema3A* or *NRP1*, we divided them into 2 patient groups (top 25% and the rest) and generated Kaplan-Meier survival curves of each group. High expression of *Sema3A* or *NRP1* was associated with poor patient prognosis in patients with LGG or GBM (Figure 9, B–D). Finally, we determined whether mRNA levels of Sema3A and NRP1 are associated with GBM subtypes assigned by the Verhaak GBM subtype classification (39). In all data sets, *NRP1* mRNA level was most significantly elevated in the mesenchymal subtype (Figure 9E). *Sema3A* expression was also high in the mesenchymal subtype, although it was not statistically significant (data not shown). Taken together, these results demonstrate that the Sema3A/NRP1 signaling axis is associated with poor prognosis, is particularly elevated in the mesenchymal subtype, and is correlated with TGF-β activity in a large glioma cohort (Figure 10 and Supplemental Figure 5).

## Discussion

This report has aimed to identify what we believe to be novel pathways that promote the growth of GSCs, which are a critical cell population that drives GBM propagation and recurrence. We found that Sema3A, originally known as an axon guidance molecule in the CNS, is a potent mitogen for GSCs but not NPCs. As a mechanistic link, we demonstrated that Sema3A binds to its receptor NRP1 and activates oncogenic TGF-β signaling through an interaction between NRP1 and TGF-βR1. The activation of Sema3A-mediated TGF-β signaling is prominent in specimens of patients with GBM, particularly in the mesenchymal GBM subtype. Based on this, we propose a protumorigenic role of the Sema3A/NRP1/TGF-βR1 signaling axis in GBM.

Recent studies have uncovered the roles of semaphorins in GBM cell proliferation and invasion. Sema3C promotes GSC survival and proliferation through activation of RAS-related C3 botulinum toxin substrate 1 or canonical Wnt pathway (16, 40). Higgins et al. reported that exogenous Sema3A administration inhibited patient-derived GBM stem cell proliferation but increased invasiveness (41). While our Sema3A-dependent proliferation data are different from those in Higgins et al., NRP1 knockdown has shown the delayed tumor growth in both studies (41). One potential explanation for this discrepancy can be attributed to the differential status of TGF-β signaling in GBM tumors. Additional factors include the differences in the experimental settings, including cell culture conditions, biological readouts, and various genomic alterations, found in patient GBMs.

During development, Sema3A forms a complex with plexins or NRP1, in which multiple plexin family proteins are implicated. Similarly, NRP1 or 2 are known to interact with multiple receptor proteins, including VEGFRs and integrin family proteins (23, 42). We did not extensively examine the involvement of the above molecules, which is a major limitation of this study. Further studies to fully characterize the roles of the other signaling players including plexins, neuropilins, and integrins are warranted. In addition, it would be informative to determine whether other Sema family proteins, including Sema3A and Sema3C, share the similar downstream effectors and activate various oncogenic signaling in GBM. Notwithstanding this caveat, our findings on the Sema3A/NRP1/TGF-βR1/SMAD2 axis suggest a potentially previously unrecognized GBM-specific oncogenic signaling.

The Sema3A/NRP1 axis contributes to promote angiogenesis, recruitment of tumor-associated macrophages, and tumor-induced immune privilege (22, 43, 44). Interestingly, NRP1 mRNA expression is particularly high in the mesenchymal GBM subtype, which is associated with the worst prognosis and elevated TGF-β pathway activity compared with other subtypes (13, 39). TGF-β pathway can also promote mesenchymal transdifferentiation, which often occurs in recurrent tumors after standard therapy to change the tumor-associated immune microenvironment. This may suggest that Sema3A/NRP1/TGF-β axis-mediated signaling elicits protumorigenic effects by regulating tumor-autonomous proliferative pathways and the tumor microenvironment (TME). Moreover, downstream effectors of TGF-β signaling are known to play central roles in the TME remodeling (45). Although oncogenic roles of TGF-β signaling have been well established and various TGF-β pharmacological inhibitors have been developed, clinical translation of these approaches remains unclear. Targeting of Sema3A or NRP1 can be an alternative therapeutic approach to inhibit oncogenic TGF-β signaling in cancer. Further studies may elucidate potential relationships between these signaling molecules in the context of the TME and cellular hierarchy.

In summary, our data provide a molecular basis of the Sema3A/NRP1/TGF-βR1 signaling axis in GBM and suggest that this signaling node is a key GSC regulator and a potential therapeutic target. Together, these data emphasize the importance of understanding the roles of various neurotransmitters, synaptic adhesion molecules, and axon guidance molecules in CNS tumor initiation and progression, which may yield new insights into the molecular mechanisms of GBM and potential therapeutic targets for this deadly disease.

## Methods

### Patient-derived GBM specimens and primary cell culture.

Patient-derived primary GBM cells and NPCs were cultured as previously described (46, 47). Briefly, GBM cells were maintained in Neurobasal medium supplemented with N2, B27, and bFGF and EGF (NBE medium: Neurobasal media, N2 and B27 supplements [0.5× each; Invitrogen], and human recombinant bFGF and EGF [25 ng/mL each; R&D Systems]) (46, 48). For recombinant protein screening assays, GBM cells were cultured in the absence of bFGF and EGF. For sphere culture, GSCs and NPCs were cultured in uncoated plastic dishes. NPCs were obtained from Lonza, Aruna, and 101Bio. Normal NPCs (Lonza, catalog PT-2599; Aruna, catalog, hNP7013; 101Bio, catalog P801) and normal human astrocytes (from Lonza) were purchased and cultured as recommended.

### Plasmids and lentiviral transduction.

Lentiviral vectors expressing shRNAs for *Sema3A*, *NRP1*, and *TGF-βRI* were purchased from MilliporeSigma. Lentiviral vectors expressing WT *NRP1* and *TGF-βR1*, an *NRP1* mutant without CUB domain (a Sema-binding domain, aa 28–265 of NRP1 protein), and T204D TGF-βR1 proteins were validated by sequencing and IB analysis. For viral production, HEK293T cells (ATCC) were cotransfected with a lentiviral expression vector and packaging plasmids (psPAX2 and pCMV-VSV-G) using CalPhos Mammalian Transfection Kit (Clontech). Virus-containing supernatants were collected and concentrated by ultracentrifugation (90,000*g* for 2 hours at 4°C). The titer of each lentivirus was determined by serial dilution.

### Orthotopic GBM xenograft models.

Six-week-old male BALB/c nude mice (Orient Bio) were used for intracranial transplantation. Patient-derived glioma cells (1 × 10^5^ per mouse) were injected into the brains of mice by stereotactic intracranial injection (coordinates: 2 mm anterior, 2 mm lateral, and 2.5 mm depth from the dura). For in vivo growth competition assays, xenograft tumor-derived GBM cells (022, 578, 609, 559, 131, and 378) were labeled with either GFP or RFP using lentiviral infection. After checking fluorescence signals through FACS analysis, GFP-labeled GBMs were infected with NT lentivirus (control) and RFP-labeled GBMs were infected with *NRP1* targeting shRNA lentivirus. GBM samples were dissociated to single cells using Accutase, and 1 × 10^5^ GBM cells were mixed with 5 μL of HBSS for 1 mouse and injected intracranially into the striatum of an adult nude mouse by using a stereotactic device (Kopf Instruments). Mice with tumor formation were sacrificed for the primary culture. Primary tumors were harvested, minced, and incubated with Collagenase (Gibco), Dispase (Gibco), and DNase I (Roche) mixture for 10 minutes. Dissociated cells were filtered through a 40 μm mesh (Thermo Fisher Scientific) and then processed for FACS analysis using LSR II Fortessa flow cytometer (BD Biosciences). The expression levels of GFP and RFP were determined using the FlowJo program.

### FACS sorting and analysis.

GBM cells were dissociated into single-cell suspensions, labeled with anti–NRP1-PE Ab (FAB3870P, R&D Systems), and sorted and analyzed by FACSAria II cell sorter (BD Biosciences). Data were collected and analyzed using FlowJo software.

### Cell proliferation assay (EdU staining).

EdU staining was performed using a Click-iT EdU imaging kit (Invitrogen) according to the manufacturer’s protocol. A total of 10 μM of EdU was added into the culture media for 2 hours, and cells were fixed with 4% paraformaldehyde in PBS. For short-term proliferation assays, 5 × 10^4^ cells were plated onto laminin-coated (20 μg/mL) 24-well plates with or without Sema3A.

### Tumorsphere forming limiting dilution assays.

Limiting dilution assay was performed in 96-well plates. Briefly, dissociated cells were seeded (1 to 200 cells per well) without FGF2 and EGF, and then 10 ng/mL Sema3A or 5 ng/mL TGF-β1 was added every 3 days. For inhibition of TGF-β pathway, 2 μM of SB-431542 (Tocris) was used. At the time of quantification, each well was examined for formation of tumor spheres. Stem cell frequency was calculated using extreme limiting dilution analysis (http://bioinf.wehi.edu.au/software/elda/).

### scRNA-Seq data analysis.

GBM cells were dissociated with Accutase and suspended in 1% BSA PBS solution. Live-cell FACS was performed with DRAQ5 fluorescent probe (62251, Thermo Fisher Scientific). Cell vitality was determined by trypan blue staining, and live cells were diluted to a final concentration of 1,000 viable cells/μL in 0.1% BSA PBS solution. scRNA-Seq library preparation and sequencing were performed as previously reported (34, 49). ScRNA-Seq data were processed through 10x Genomics Chromium Single Cell Platform, and count matrices were generated using the Cell Ranger pipeline (10x Genomics). Unbiased clustering was performed by uniform manifold approximation and projection dimensionality reduction visualization analysis (34). Gene signature sets used in this report are GBM subtype (39), stemness (32, 36), and TGF-β signatures (33). All single-cell profiles can be downloaded publicly (National Center for Biotechnology Information Gene Expression Omnibus [NCBI GEO] GSE162931).

### Quantitative real-time reverse transcription PCR.

Real-time PCR was performed on an ABI Prism 7900 sequence detection system (Applied Biosystems) according to the manufacturer’s instructions using the following primers: *Sox2*, Forward: 5-TGCGAGCGCTGCACAT-3, Reverse: 5-TCATGAGCGTCTTGGTTTTCC-3; *Sox4*, Forward: 5-CTGCGCCTCAAGCACATG-3, Reverse: 5-TTCTTCCTGGGCCGGTACT-3; *LIF*, Forward: 5-TCTTGGCGGCAGGAGTTG-3, Reverse: 5-CCGCCCCATGTTTCCA-3; *ID1*, Forward: 5-CTACGACATGAACGGCTGTTACTC-3, Reverse: 5-CTTGCTCACCTTGCGGTTCT-3; *ID3*, Forward: 5-TCAGCTTAGCCAGGTGGAAATC-3, Reverse: 5-TGGCTCGGCCAGGACTAC-3.

All samples including no-template controls were assayed in triplicates. Quantification of target gene expression was performed with comparative threshold cycle method.

### IP and Western blot assays.

Cells were lysed in Pierce IP lysis buffer (Thermo Fisher Scientific) supplemented with proteinase inhibitor and phosphatase inhibitor. Blots were incubated with rabbit anti-NRP1 (catalog ab81321, Abcam), rabbit anti–TGF-βRI (catalog sc-402, Santa Cruz Biotechnology), goat anti-Sema3A (catalog sc-1147, Santa Cruz Biotechnology), rabbit anti-SMAD2 (catalog 5339, Cell Signaling Technology), rabbit anti–phospho-SMAD2 (catalog 3108, Cell Signaling Technology), rabbit anti-AKT (catalog 4691, Cell Signaling Technology), rabbit anti–phospho-AKT (catalog 4060, Cell Signaling Technology), rabbit anti-VEGFR2 (catalog 9698, Cell Signaling Technology), rabbit anti–phospho-VEGFR2 (catalog 3770, Cell Signaling Technology), mouse anti-V5 (catalog MA1-34099, Invitrogen), and mouse anti-Actin (catalog sc-8432, Santa Cruz Biotechnology) overnight at 4°C. For IP, 500 μg of proteins were isolated from GBM cells treated with or without Sema3A or VEGF (R&D Systems), incubated overnight with 2 μg of NRP1 or TGF-βR1 Ab, conjugated to protein A/G beads (catalog sc-2003, Santa Cruz Biotechnology), washed, and then separated on SDS-PAGE gels.

### PLA.

Cells were treated with rSema3A or vehicle control for 10 minutes and fixed with 4% paraformaldehyde for 15 minutes. Tumor sphere sections were blocked with 5% goat serum in PBS containing 0.2% Triton X-100 and incubated overnight with Abs against anti-NRP1 (catalog sc-7239, 1:250; Santa Cruz Biotechnology) and anti–TGF-βR1 (catalog sc-402, 1:250; Santa Cruz Biotechnology). The proximity ligation reaction and visualization of the signal were performed according to the manufacturer’s protocol using the Duolink Detection Kit with PLA PLUS and MINUS probes for mouse and rabbit Abs (catalog DUO92004 and DUO92002, Sigma-Aldrich). DAPI stain was used to detect cell nuclei. Alexa Fluor 488 phalloidin (Life Technologies) was used to visualize actin cytoskeleton in cells.

### TMA and immunostaining analysis.

Paraffin-embedded TMAs were constructed at the Samsung Medical Center. TMA slides include10 non-neoplastic brain tissues and 68 GBM specimens. After blocking and permeabilization with 0.3% Triton X-100 and 10% goat or donkey serum in PBS, tissue sections were probed with the following primary Abs: rabbit anti-NRP1 (catalog ab81321, Abcam), goat anti-Sema3A (catalog sc-1147, Santa Cruz Biotechnology), rabbit anti-ID1 (catalog sc-133104, Santa Cruz Biotechnology), goat anti-Sox2 (catalog AF2018, R&D Systems), rabbit anti–phospho-SMAD2 (catalog 3108, Cell Signaling Technology), and rabbit anti–glial fibrillary acidic protein (catalog GA524, DAKO). Appropriate fluorescence-tagged secondary Abs and HRP-conjugated Abs were used for visualization: Donkey anti-Goat IgG antibody (H+L), Alexa Fluor 594 (catalog A-11058, Invitrogen); Donkey anti-Rabbit IgG antibody (H+L), Alexa Fluor 594 (catalog A-21207, Invitrogen); Donkey anti-Rabbit IgG antibody (H+L), Alexa Fluor 488 (catalog A-21206, Invitrogen); Rabbit anti-goat IgG antibody (H+L), Biotinylated (catalog BA5000, Vector Laboratories); Goat anti-Rabbit IgG antibody (H+L), Biotinylated (catalog BA1000, Vector Laboratories); Goat anti-Rabbit IgG antibody (H+L), HRP (catalog 31460, Invitrogen); Goat anti-Mouse IgG antibody (H+L), HRP (catalog 31430, Invitrogen); and Rabbit anti-Goat IgG (H+L) antibody, HRP (catalog 31402, Invitrogen). Images were taken by Leica DM4000B microscope and analyzed by IHC Profilter. IF images were taken by a Leica TCS SP5 Confocal Microscope. To ensure unbiased quantitation of the results, we employed a double-blind protocol in which the scientists who performed the immunostaining and technicians who evaluated the staining intensities did not know the sample information.

### Bioinformatics data analysis.

The REMBRANDT (50) and TCGA databases (37, 38) were used to analyze correlations between mRNA expression, patient survival, glioma subtypes, genetic alterations, and TGF-β activity (33).

### Statistics.

All data were expressed as mean ± SD from at least 3 independent experiments. Quantification of immunopositive cells in immunostaining analyses was carried out using NIH ImageJ software (http://rsb.info.nih.gov/nih-image/). For the animal survival studies, *P* values were determined by log-rank test. One-way ANOVA, 1-way ANOVA with Tukey’s multiple-comparison test, 2-way ANOVA, Mann-Whitney test, pairwise *t* test, and Student’s 1-tailed *t* test were used to determine statistical significance. We consider *P* values less than 0.05 as significant.

### Study approval.

Tumor samples classified as GBM, based on WHO criteria, were obtained from patients undergoing surgical treatment in accordance with the NIH, Cleveland Clinic Lerner Research Institute, and Samsung Medical Center Institutional Review Boards. All mouse experiments were performed according to the guidelines of the Animal Use and Care Committees at the Samsung Medical Center and Association for Assessment and Accreditation of Laboratory Animal Care–accredited guidelines.

### Data availability.

All data are contained within the manuscript or supplemental materials. All raw data can be accessed in the supplement and Supporting Data Values file. scRNA-Seq data are publicly available in the NCBI GEO database (GSE162931). Further information supporting the findings is available upon request from the corresponding author.

## Author contributions

HMJ, DHN, and JL conceived the overall research design. HMJ, BWP, and JL mainly wrote and edited the manuscript. HMJ, YJS, JHL, NC, DHW, WK, HJC, HY, JKL, YL, and DGK conducted experiments and acquired data. WJL, DN, HJC, and JKS performed bioinformatics analyses. BWP, DHN, and YY provided reagents and intelligent inputs throughout the research. HMJ is listed before YJS based on her primary contribution in the research design and manuscript writing.

## Supplementary Material

Supplemental data

Supporting data values

## Version 1 10/03/2023

In-Press Preview

## Version 2 11/08/2023

Electronic publication
