# Supplementary material for: The semaphorin 3A/neuropilin-1 pathway promotes clonogenic growth of glioblastoma via activation of TGF-β signaling
Source: JCI Insight. 2023 Nov 8;8(21):e167049. doi: 10.1172/jci.insight.167049 (PMC10721275; doi:10.1172/jci.insight.167049)
Supplement: Supplemental data [file jciinsight-8-167049-s044.pdf]

Supplemental Information

Supplemental table 1: A mitogen screen using GSCs and NPCs

	131		827		NPC1		NPC2	
	Mean	SD	Mean	SD	Mean	SD	Mean	SD
untreated control	5.389	0.180	4.744	0.068	0.812	0.204	0.750	0.204
EGF	81.417	2.242	65.917	1.083	9.917	0.110	6.833	1.167
bFGF	23.890	1.800	28.667	2.075	4.375	0.552	6.292	0.150
EGF+bFGF	111.540	5.342	74.322	4.342	12.460	2.445	8.984	1.452
HGF	27.958	1.522	12.389	0.180	3.240	0.667	2.873	0.331
beta-NGF	8.625	0.580	9.556	0.515	2.528	0.778	3.222	0.068
NT-3	5.500	0.637	3.472	0.646	0.760	0.177	0.783	0.114
BDNF	6.708	1.017	4.583	0.454	1.958	0.184	2.528	0.148
Semaphorin 3A	27.250	3.146	14.958	2.765	0.775	0.125	0.317	0.218
Semaphorin 3B	3.486	1.195	5.875	1.816	0.867	0.326	1.083	0.505
Semaphorin 3C	9.292	5.745	6.463	2.253	1.153	0.417	0.992	0.126
Semaphorin 3D	5.917	1.134	2.992	1.125	0.750	0.250	0.708	0.260
Semaphorin 3E	3.494	1.706	2.875	1.192	0.708	0.191	0.861	0.427
Semaphorin 5A	8.861	3.071	7.250	3.279	0.725	0.214	0.917	0.391
Semaphorin 6A	7.069	1.209	5.069	1.916	0.883	0.347	1.069	0.295
Semaphorin 6D	2.403	1.144	3.403	0.690	0.583	0.144	0.708	0.191
Slit3	5.917	0.813	4.617	0.586	0.750	0.250	0.792	0.315
Slit1	5.733	1.783	4.408	0.396	0.875	0.125	0.833	0.382
RGM-B	3.319	0.856	4.580	0.640	0.875	0.139	0.817	0.388
RGM-A	3.528	0.937	3.403	0.945	0.708	0.191	0.683	0.161
Ephrin-A1	3.611	2.014	3.992	0.647	0.775	0.115	0.583	0.629
Ephrin-A5	5.319	2.341	4.792	0.688	0.808	0.170	0.417	0.382
Ephrin-B2	8.653	3.623	5.125	4.823	0.692	0.201	0.708	0.191
Ephrin-B1	7.833	3.001	8.667	3.711	0.708	0.191	0.667	0.289
Neuroligin 1	4.986	0.307	5.075	0.385	0.833	0.315	1.083	0.520
Neuroligin 2	4.528	1.100	6.875	1.231	0.733	0.225	1.542	0.711
Neuroligin 3	11.861	1.737	7.542	2.688	1.336	0.604	0.950	0.189
Nogo-A	7.319	2.260	6.833	3.138	0.783	0.125	0.686	0.681
Omgp	6.490	1.593	5.235	0.936	0.828	0.423	0.932	0.782

We chose 24 recombinant proteins that are critical regulators of synaptic adhesion, axon guidance, and neurotropic functions. Both GBM cells and NPCs were plated at varying seeding densities (5 to 200 cells per well) in serum-free media and then their growth was evaluated two weeks later by counting the number of resultant spheres.

Supplemental figure legends

Figure S1. Sema3A induces GBM cell proliferation via NRP1.

(A) LDA analysis to determine clonogenic growth of GBM cells (131, 387 and 559) treated with or without rSema3A. Varying number of cells (1 to 100) was seeded into the each well (30 per each condition) and allowed to grow. Wells without sphere formation were counted. *p < 0.01 by pairwise t test.

(B) LDA analysis to determine clonogenic growth of GSCs (131, 387, 83) with *NRP1* shRNA or with NT shRNA. *p < 0.01 by pairwise t test.

Figure S2. The Sema3A-NRP1-TGFβRI signaling axis in GBM

(A and B) Co-immunoprecipitation (Co-IP) analysis of NRP1 and TGFβR1 in patient derived GBM tumors (047, 050) and the derivative cells (131, 559, 83). IgG represents a control antibody used for IPs.

(C) Immunoblots of pSMAD2 and total SMAD2 expression in patient derived GBM cells (559 and 047) and NPCs treated with or without rSema3A.

(D) Co-IP analyses of NRP1 (WT), NRP1 mutant (MT), and TGFβR1 proteins in 387 GBM cells treated with rSema3A. IgG represents a control antibody used for IPs.

(E) Immunoblots of pSMAD2 and total SMAD2 expression in V5-tagged wild type *NRP1* (WT) and *NRP1* mutant (MT) treated with or without rSema3A.

(F) Short-term proliferation analysis of 387 GBM cells overexpressing the control vector, wild type *NRP1*, or *NRP1* mutant vector. These cells were treated with or without rSema3A. n = 3. Data represent mean ± SD. *p < 0.01 by one-way ANOVA with Tukey's multiple comparison test in F. NS represents non-significant.

Figure S3. Characterization of TGFβRI-T204D protein

(A) Immunoblots of TGFβRI, pSMAD2 and SMAD2 in GBM cells expressing a constitutively active TGFβ type I receptor (*TGFβRI-T204D*).

(B) Short-term proliferation analysis of 131 and 559 GBM cells expressing TGFβRI-T204D protein. n = 3. Data represent mean ± SD. *p < 0.01 by one-way ANOVA. NS represents non-significant.

(C) LDA analysis of 131 and 559 GBM cells expressing TGFβRI-T204D protein. *p < 0.01 by pairwise t test.

Figure S4. Correlations between the expression levels of *NRP1* and *Sema3A* or *TGFRβ1* in LGG/GBM specimens*.*

In silico analyses showing positive correlations between *NRP1* and *Sema3A* or *TGFRβ1* (n=667). r values were calculated by Pearson's correlation test. All analyses were performed using GlioVis (http://gliovis.bioinfo.cnio.es/) (1).

Figure S5. Correlation between *NRP1* mRNA levels and TGFβ activity in gliomas.

(A) Heat map plot of glioma subtypes and TGFβ activity based on the *NRP1* mRNA levels.

(B) Correlation between *NRP1* mRNA and TGFβ activity in gliomas. n = 157. r values were calculated by Pearson's correlation test. *p < 0.00001

Reference

1. Bowman RL, Wang Q, Carro A, Verhaak RG, and Squatrito M. GlioVis data portal for visualization and analysis of brain tumor expression datasets. *Neuro Oncol.* 2017;19(1):139-41.

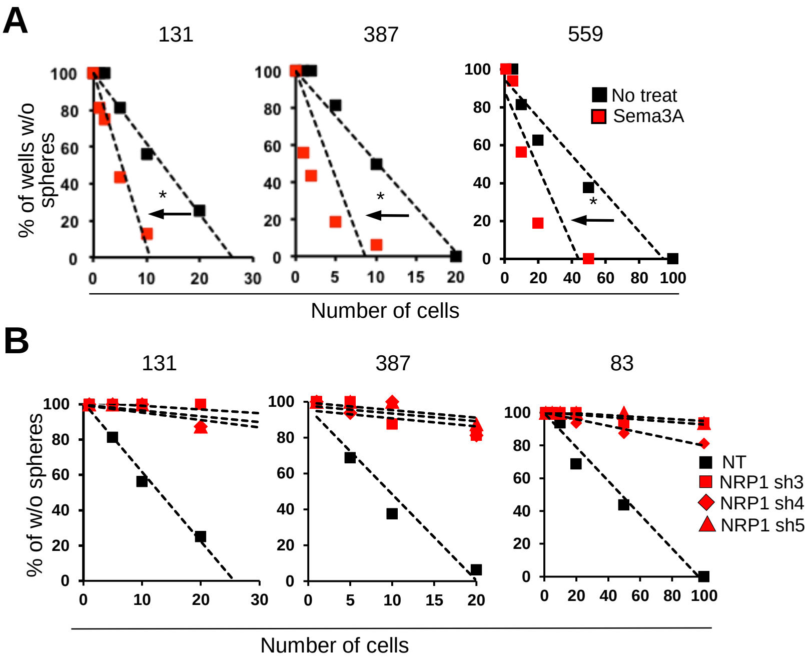
A
131
387
559
% of wells w/o spheres
No treat
Sema3A
Number of cells
B
131
387
83
% of w/o spheres
NT
NRP1 sh3
NRP1 sh4
NRP1 sh5
Number of cells

Figure S1

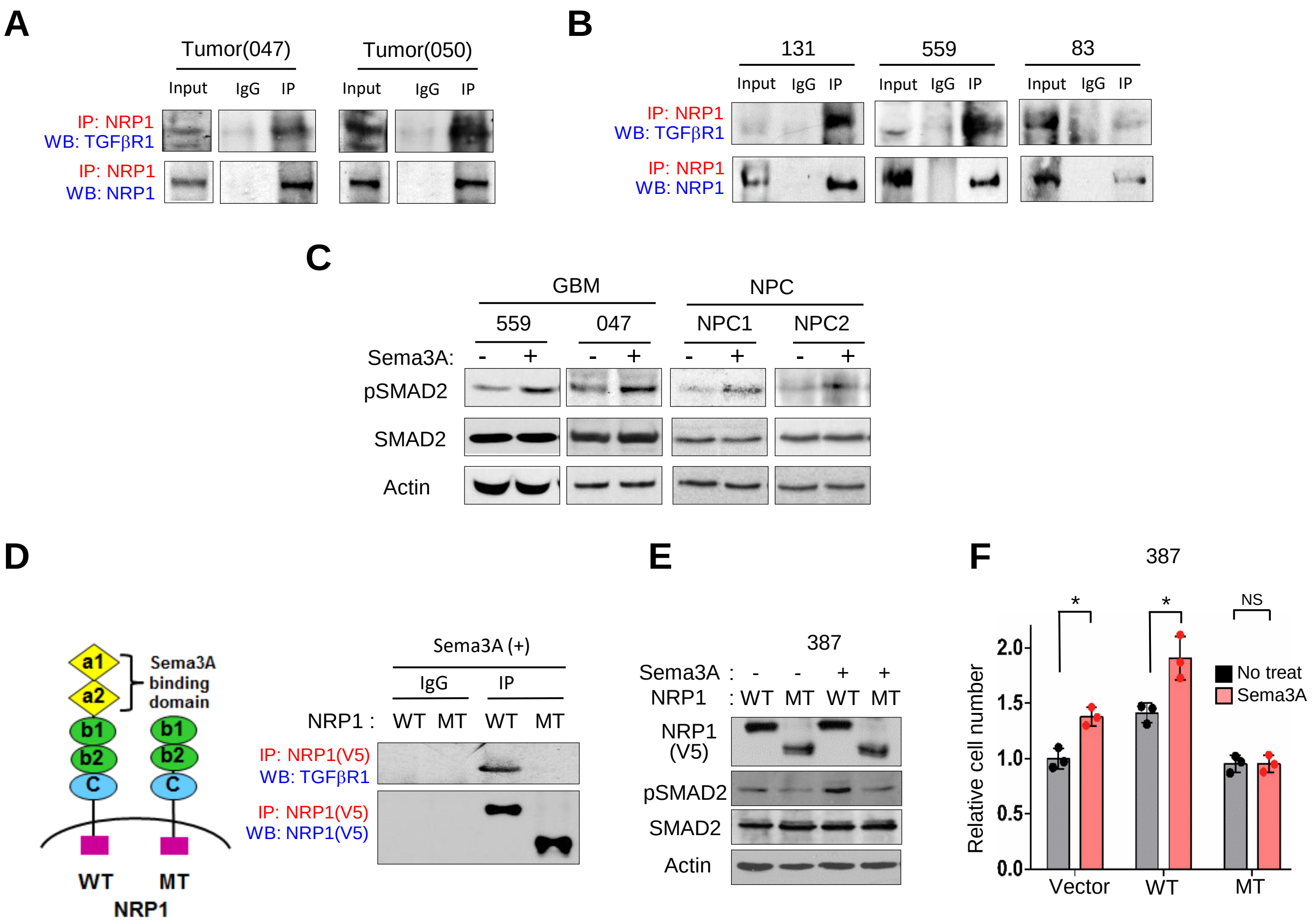
A
Tumor(047)
Tumor(050)
Input
IgG
IP
IP: NRP1
WB: TGFβR1
IP: NRP1
WB: NRP1
B
131
559
83
C
GBM
NPC
559
047
NPC1
NPC2
Sema3A:
pSMAD2
SMAD2
Actin
D
a1
a2
Sema3A binding domain
b1
b2
C
WT
MT
NRP1
Sema3A (+)
NRP1 :
IP: NRP1(V5)
WB: TGFβR1
IP: NRP1(V5)
WB: NRP1(V5)
E
387
Sema3A :
NRP1 :
NRP1 (V5)
F
387
Relative cell number
No treat
Sema3A
Vector
NS
*
0.0
0.5
1.0
1.5
2.0

Figure S2

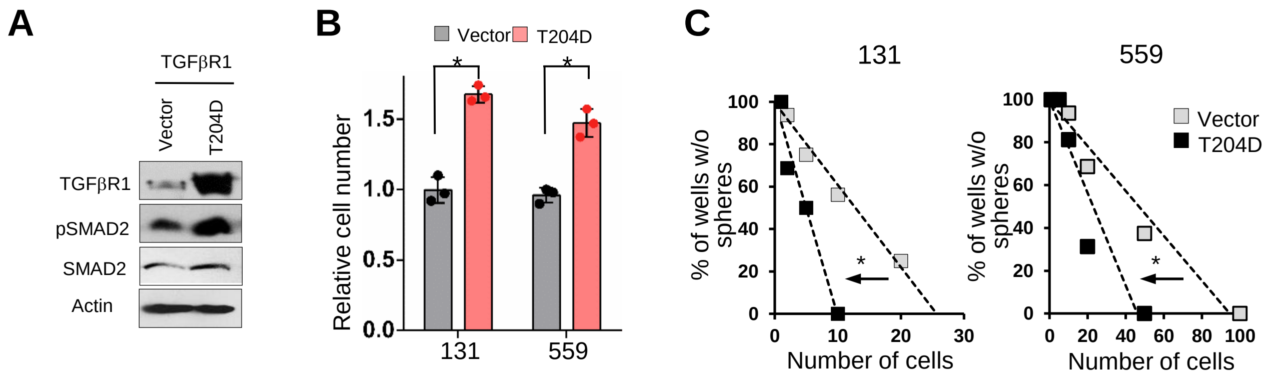
A
TGFβR1
Vector
T204D
TGFβR1
pSMAD2
SMAD2
Actin
B
Vector
T204D
*
*
Relative cell number
0.0
0.5
1.0
1.5
131
559
C
131
559
% of wells w/o spheres
Number of cells
Vector
T204D
*
*

Figure S3

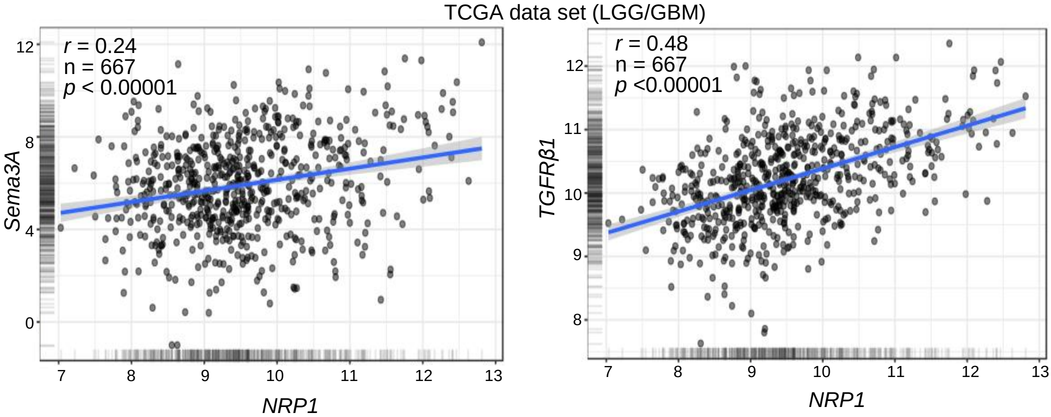

TCGA data set (LGG/GBM)
r = 0.24
n = 667
p < 0.00001
Sema3A
NRP1
12
8
4
0
7
8
9
10
11
12
13
r = 0.48
n = 667
p <0.00001
TGFRβ1
NRP1
12
11
10
9
8
7
8
9
10
11
12
13

Figure S4

A

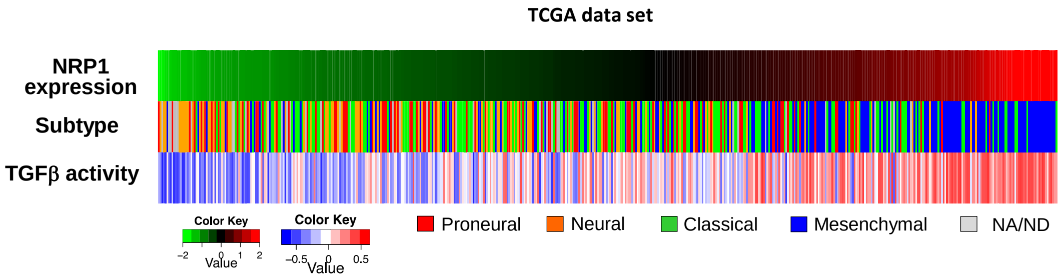
TCGA data set
NRP1 expression
Subtype
TGFβ activity
Color Key
−2
0
1
2
Value
Color Key
−0.5
0
0.5
Value
Proneural
Neural
Classical
Mesenchymal
NA/ND

B

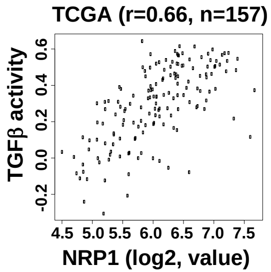
TCGA (r=0.66, n=157)
TGFβ activity
-0.2
0.0
0.2
0.4
0.6
4.5
5.0
5.5
6.0
6.5
7.0
7.5
NRP1 (log2, value)

Figure S5
